# Towards standardized automated immunomonitoring: an automated ELISpot assay for safe and parallelized functionality analysis of immune cells

**DOI:** 10.1007/s10616-016-0037-4

**Published:** 2016-11-28

**Authors:** J. C. Neubauer, I. Sébastien, A. Germann, S. C. Müller, A. Meyerhans, H. von Briesen, H. Zimmermann

**Affiliations:** 1 Department of Medical Biotechnology, Fraunhofer Institute for Biomedical Engineering, Sulzbach, Germany; 2 Infection Biology Laboratory, Department of Experimental and Health Sciences, Universitat Pompeu Fabra, Barcelona, Spain; 3 Institució Catalana de Recerca i Estudis Avançats (ICREA), Barcelona, Spain; 4 Department of Molecular and Cellular Biotechnology, Saarland University, Saarbrücken, Germany

**Keywords:** ELISpot, Automation, Liquid classes, Operator safety, HIV vaccines, PBMC

## Abstract

The ELISpot assay is used for the detection of T cell responses in clinical trials and vaccine evaluations. Standardization and reproducibility are necessary to compare the results worldwide, inter- and intra-assay variability being critical factors. To assure operator safety as well as high-quality experiment performance, the ELISpot assay was implemented on an automated liquid handling platform, a Tecan Freedom EVO. After validation of the liquid handling, automated loading of plates with cells and reagents was investigated. With step by step implementation of the manual procedure and liquid dispensing optimization on the robot platform, a fully automated ELISpot assay was accomplished with plates remaining in the system from the plate blocking step to spot development. The mean delta difference amounted to a maximum of 6%, and the mean dispersion was smaller than in the manual assay. Taken together, we achieved with this system not only a lower personnel attendance but also higher throughput and a more precise and parallelized analysis. This platform has the potential to guarantee validated, safe, fast, reproducible and cost-efficient immunological and toxicological assays in the future.

## Introduction

Developed first in 1983 to detect antibody secreting cells (Czerkinsky et al. 1983), the enzyme linked Immunospot (ELISpot) assay improved continually and gained more and more attention over the years (Czerkinsky et al. 1988). With the recommendation of the 13th International AIDS Congress in 2000 to use the ELISpot technique due to its performance for immunomonitoring purposes, it became one of the most important ex vivo methods in cellular immunology (Janetzki 2004). Based on the ELISA technique, the ELISpot assay is used worldwide in diverse areas ranging from vaccinology and tumorology to infectious diseases and transplantation (Almeida et al. 2009). Besides the detection, measurement and characterization of immune cell activities in clinical and cancer trials (Cox et al. 2006), it helps to evaluate new vaccines in order to control and prevent infectious diseases such as those caused by *mycobacterium tuberculosis* (Bathoorn et al. 2011; Kobashi et al. 2010), hepatitis-C-virus and HIV (Lee et al. 1989).

Despite this potential, inter-operator and inter-assay inconsistency of the measurement is one of the most critical limitations of the method (Janetzki 2004). As clinical trials require comparability and reproducibility of results, the ELISpot assay therefore should be more simple and accurate to become a standardized and validated method. The first steps to harmonize the process for industrial applications included standardization of protocols, materials and reagents e.g. by using pre-coated 96 well plates (Cox et al. 2006) and well-defined antibodies and enzymes (Janetzki 2004). As shown by the Cancer Vaccine Consortium (CVC), protocol choices and laboratory practices can have dramatic effects on assay performance (Janetzki et al. 2008). The CVC described in a guideline the recommendations for successful assay outcome such as established laboratory standard operation procedures (SOP), counting method, cell preparation, serum quality and spot evaluation. These conditions result from two ELISpot proficiency panels initiated in 2005 which were aimed to identify deficient practices and common sources of variability between laboratories to increase standardization (Janetzki et al. 2008). One complementary approach to improve the assay was the introduction of automation along the process as described in Janetzki et al. (2004). Two automated machines had particularly great impact on the standardization and simplification of the ELISpot assay. First, the introduction of automated cell counters on the market permitted comparable viable starting cell concentrations in all laboratories without individual cell counting and method variability. Second, the implementation of automated ELISpot readers (Hawkins et al. 2006; Zadorozhny and Martynov 2012) allowed the evaluation of assay results by counting developed spots automatically. This option offers a rapid and compliant assessment with lower variability than manual spot counting on a stereomicroscope. Despite this progress of automation, the ELISpot assay is still a very error-prone method depending on many parameters like cell cryopreservation (Maecker et al. 2005), reagent manufacturer, incubation times, spot evaluation (Janetzki et al. 2004), analysis criteria (Janetzki 2004; Moodie et al. 2012) and operator pipetting accuracy (Almeida et al. 2009; Maecker et al. 2008).

Our challenge in this study was to reduce these detrimental influences by developing an automated ELISpot assay. The aim was to minimize the human factor on inter-operator, inter-assay and intra-assay variability and optimize precision and reproducibility (Janetzki et al. 2008). An automated system that employs robotic technology to control processes or to achieve automatic operations without human intervention has many advantages. It allows high reproducibility and result accuracy combined with a high throughput of data through parallelization and scheduling, a continuous and safe electronic control of the process with data recording, a non-stop working time and above all, cost-efficiency with a low need of personnel. All these performance indicators increased the interest in automated platform development in the last decade (Ferreira et al. 2011; Jiang et al. 2012; Leguia et al. 2011; Sarkozi et al. 2003) and fulfilled many expectations of the pharmaceutical industry in search of GCLP-validated high throughput screening systems. Various platforms have recently been developed to be used as closed and sterile systems, integrating all necessary biological devices for cell culture and maintenance (Koike et al. 2012; McLaren et al. 2013) or pseudovirus production (Schultz et al. 2012). First tests of an automated ELISpot assay have been performed with a simple liquid handling robot combined with a cell counter, as well as a plate washer and reader by Almeida et al. (2009). The system described by us here allows, in addition, the complete integration and automation of cell culture and incubation steps, so that plates remain in the system from the plate blocking step to spot development.

Based on our expertise in implementing and validating an automated system under GCLP, we (1) show a technical way to improve an automated immunomonitoring tool on a pipette robot platform and (2) define which parameters have to be considered, characterized and adapted. In this paper we focus on the automation of the ELISpot assay due to the infectious potential of the assay and the necessity to reduce the risk for the personnel. At the end of the process optimization we demonstrate the feasibility of running a safe assay and obtain precise results, in a short time. This assembly of several automated steps in a higher process minimizes dangerous contacts of the laboratory staff with infectious cell samples by realizing the process in a closed, safe and sterile environment. At the same time, a higher sample number can be tested with better accuracy and lower personnel attendance. This setup is suitable for laboratories in which highly pathogenic microbes are handled and operator protection has to be guaranteed. In addition, the platform could be used for a centralized evaluation of large vaccination multicenter studies to avoid laboratory and operator dependency and to provide the highest possible results comparability.

## Material and method

### Blood cells isolation and cryopreservation

Citrated buffy coats were provided by the blood donor center “Blutspendezentrale Saar-Pfalz gGmbH Am Klinikum Saarbrücken” in Saarbrücken (URL: http://www.blutspendezentrale-saarpfalz.de/index.html) with written informed consent of the donors (for research purposes). According to German national regulations, blood donor centers do not necessitate specific ethics statement for blood collections (Germann et al. 2013). The lymphocytes were separated from the erythrocytes with the Ficoll solution LSM 1077 (PAA, Cölbe, Germany). After a first centrifugation step (2000 rpm, 30 min, without brake) the peripheral blood mononuclear cells (PBMC) interphase layer was collected and washed once with phosphate-buffered saline solution (PBS) (Gibco, Darmstadt, Germany). After measurement of cell concentration and viability on the automated cell analyzer Vi-Cell XR (Beckman Coulter, Krefeld, Germany), PBMCs were resuspended in 10 ml R10 medium with a concentration of 1 × 10^6^ cells/ml and cultured overnight (37 °C, 5% CO_2_) for direct use in the ELISpot assay. R10 medium contains RPMI 1640 (Gibco, Darmstadt, Germany), 10% fetal bovine serum (FBS) (PAA, Cölbe, Germany), 1% Glutamax (Invitrogen, Darmstadt, Germany), 1% Penicillin/Streptomycin (PAA, Cölbe, Germany) and 2.5% 1 M HEPES (Gibco, Darmstadt, Germany). Alternatively, 1 × 10^7^ cells/ml PBMCs were frozen with serum-free GHRC cryomedium (Schulz et al. 2012) for subsequent experiments and thawed one day before the ELISpot plate loading. Cryovials were held in 37 °C water bath until a small ice crystal remained. Then 1 ml warm R10 medium was slowly added to the vial and the thawed cell suspension was directly transferred into 8 ml R10 medium. To avoid toxic effects of dimethylsulfoxid (DMSO) still present in the cell suspension, PBMCs were centrifuged for 10 min with 400 g and the pellet was re-suspended in 10 ml R10 medium. Cells rest overnight in the incubator at 37 °C and 5% CO_2_.

### ELISpot assay

**Fig. 1:**
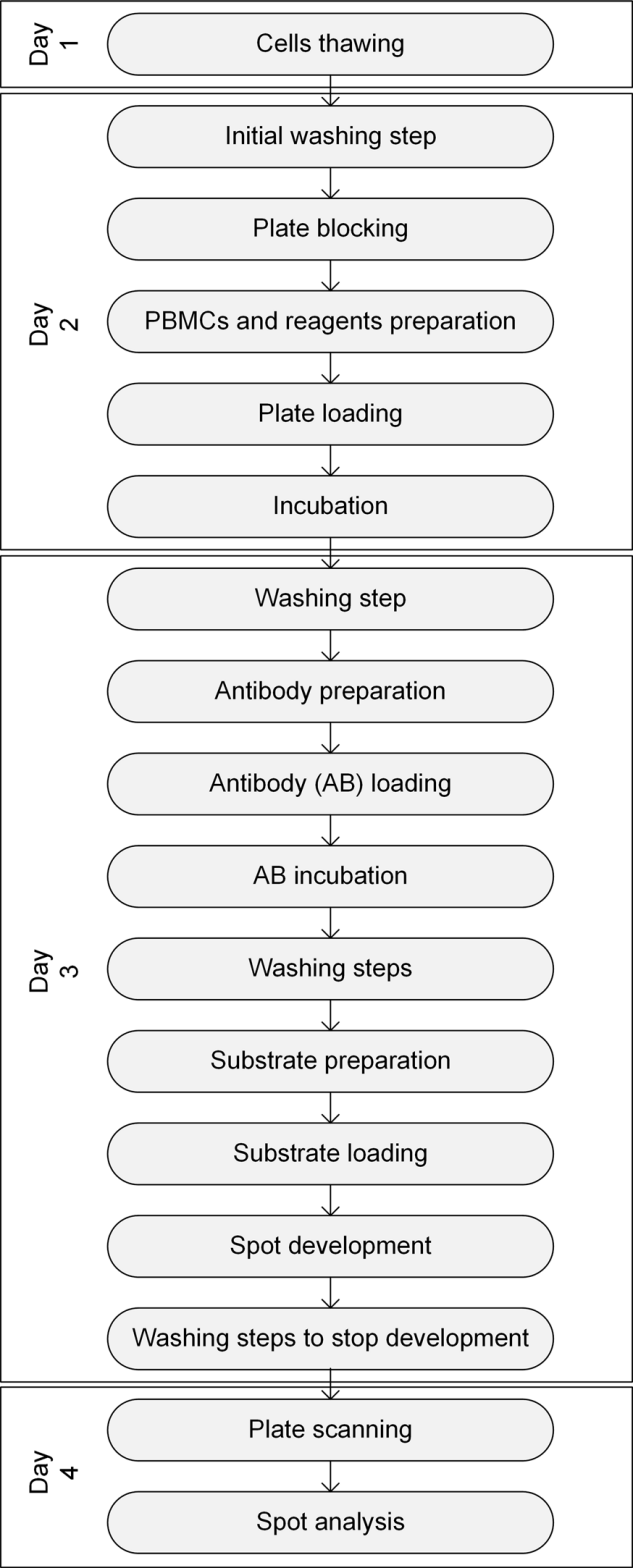
Workflow of the ELISpot assay steps over 4 days from cell preparation to plate analysis

Figure 1 illustrates the step by step workflow of an ELISpot assay from cell thawing to substrate development, as described here. The ELISpot assay requires an antibody-coated 96 well plate (anti-IFN-gamma mAb 1 D1k pre-coated, Mabtech, Nacka Strand, Sweden), prewashed with PBS (Gibco, Darmstadt, Germany) and blocked with R10 medium. After at least 30 min blocking time, 50 μl of a specific CEF (cytomegalovirus, Epstein–Barr virus, influenza virus) peptide pool, CMV (cytomegalovirus) peptide pool and PHA (phytohemagglutinin), respectively, were added per well. The samples were run in triplicates. R10 medium was used as negative control. The final stimulant concentration per well was 2 μg/ml for the CEF Peptide Pool (CTL, Bonn, Germany), 1 μg/ml for CMV (PepMix HCMVA pp65, JPT, Berlin, Germany) and 4 μg/ml for the lectin PHA (Sigma, Taufkirchen, Germany). 50 μl cell suspension with a concentration of 2 × 10^6^ cells/ml was added to each well with wide bore tips (MBP 200G, VWR, Darmstadt, Germany). After loading, the plate was stored for 20–24 h in the incubator at 37 °C and 5% CO_2_ and agitation was avoided to enable binding of secreted cytokines to the coated membrane.

On the next day, 50 μl/well detector antibody solution was added after a PBS washing step for cytokine detection. The solution consisted of a 1:200 dilution of antibody (anti-human IFN-gamma Detector antibody 7B6-1 HRP conjugated, Mabtech, Nacka Strand, Sweden) in sterile and filtered PBS containing 0.5% FBS. After 2 h of incubation time, unbound antibodies were removed by an automated washer (HydroFlex, Tecan, Crailsheim, Germany) and 50 μl/well substrate (Nova Red, Biozol, Eching, Germany) were incubated for 5 min. The reaction was stopped and the plate was dried overnight. Each developed spot corresponded to a cytokine producing-cell. The plate was finally scanned and automatically evaluated using an ELISpot Analyser from CTL (Bonn, Germany) and its ImmunoScan and ImmunoSpot software. Different parameters (Dittrich and Lehmann 2012) were configured such as the spot separation (set to 0), the minimal manual gating (set to 0.0041 mm^2^) and the counted area (set to 95%). Results were represented in graphs with the donors and antigens as x-axis and the amount of spot (Spot Forming Cells SFC/10^6^ PBMCs) as y-axis.

### Determination of delta difference, coefficient of variation and dispersion

For the comparison of manual and automated pipetting during the gravimetrical analysis, we computed with n = 48 for the fixed tips and n = 24 for the disposable tips:

- the coefficient of variation (*CV*_*pipetting*_) to analyze the precision and repeatability of the conducted experiment, according to:

1 $$CV_{pipetting} = \frac{SD}{{\bar{X}}} \times 100$$

with $SD$ for standard deviation and $\bar{X}$ for the mean volume

- the delta difference ($\Delta_{pipetting}$) to evaluate the accuracy of the liquid distribution, according to:

2 $$\Delta_{pipetting} = \left| {\frac{X(set) - X(measured)}{X(set)}} \right| \times 100$$

where $X(set)$ represents the required pipetting volume and $X(measured)$ the actual detected.

In consequence, the CV represents the extent of variability in relation to the mean volume, while the delta difference describes the dispersion of the results in comparison to the reference value.

To implement the automated ELISpot assay, we determined different parameters to evaluate the spot forming cells (SFC) on developed assays:

- the coefficient of variation (*CV*_*spot*_) to evaluate the precision and repeatability of the tips, according to:

3 $$CV_{spot} = \frac{SD}{X} \times 100$$

with $SD$ for standard deviation and $X$ for the spot count with n = 71 delta difference values for the pipetting accuracy test (W-Form). These values are based on the 12 PHA and CEF spot counts of three plates minus one outlier.

- the delta difference ($\Delta_{spot}$ and $\Delta$) to evaluate the accuracy of the automated spot counts, according to:

4 $$\Delta_{spot} = \frac{X(automated) - X(manual)}{X(manual)} \times 100$$

where $X(automated)$ represents the spot count of the automated assay and $X(manual)$ the spot count of the manual assay with n = 71 values during the pipetting accuracy test (W-Form).

5 $$\Delta = \left| {\frac{{\bar{X}(automated) - \bar{X}(manual)}}{{\bar{X}(manual)}}} \right| \times 100$$

where $\bar{X}(automated)$ represents the mean of the automated values and $\bar{X}(manual)$ refers to the mean manual ones, respectively. During the comparison of the automated and manual assays, each sample was measured in triplicates with n = 3 for the conducted Tests 1, 2, 3 and 4.

- the variance ($Var$) and dispersion ($Dis$) to show the distribution of the spot count normalized to the mean, according to:

6 $$Var = \frac{1}{n}\sum\limits_{i = 1}^{n} {\mathop {(SFC_{i} - \bar{X})}\nolimits^{2} }$$

7 $$Dis = \frac{Var}{{\bar{X}}} = \frac{1}{{n \times \bar{X}}}\sum\limits_{i = 1}^{n} {\mathop {(SFC_{i} - \bar{X})}\nolimits^{2} }$$

where $SFC_{i}$ represents the spot count and $\bar{X}$ refers to the mean value with n = 3 for the conducted Tests 1, 2, 3 and 4.

Test 1 to Test 4 compared manual and automated assays, based on the parameters Δ and Dis. The Δ value represents the relative change between the manual and the automated assay, while the dispersion refers to the spreading or distribution of the samples. The smaller these values are, the more similar are both assays.

To compare the manual process with the automated one, the Wilcoxon signed-rank test was additionally used. In this case, each well measurement was considered as a single value with *p* > 0.05 as a significance limit. Values with the CEF peptide pool and the CMV peptide pool were calculated separately, resulting in n = 12 (Test 1–Test 4).

### Pipette robot assembly

**Fig. 2:**
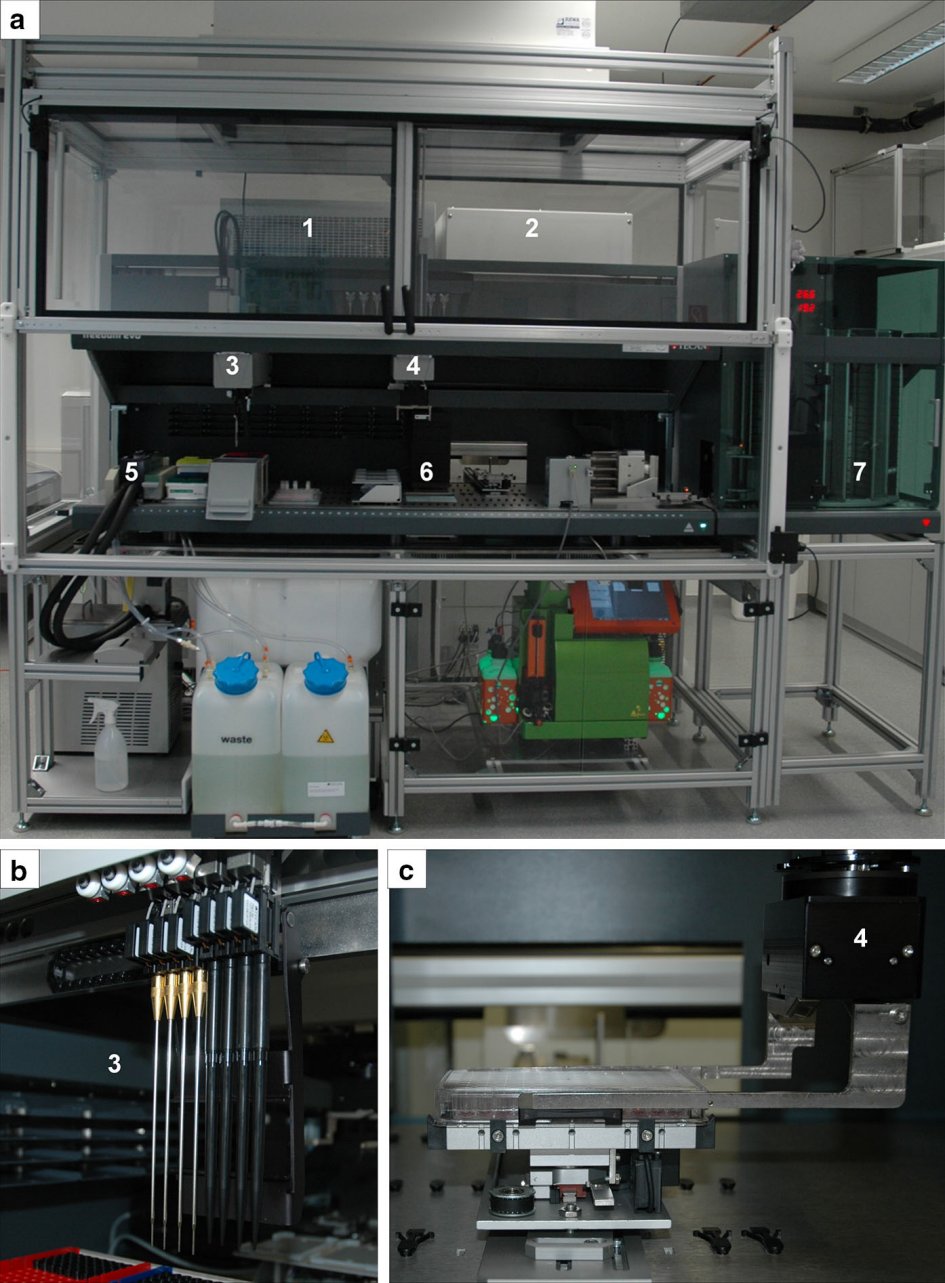
Automated pipetting platform Tecan Freedom EVO. **a** Whole system with incubator, carousel, cell counter and laminar flow amongst other devices. **b** LiHa (Liquid Handling Arm) including the four fixed needles and the four needles with single-use tips. **c** RoMA (Robot Manipulator Arm). Cell culture devices are numbered: (1) Refrigerator, (2) Incubator, (3) LiHa, (4) RoMa, (5) Wash station, (6) Dark room incubator, (7) Carousel

The liquid-handling robot platform Tecan Freedom EVO 200 consisted of different cell culture modules (Fig. 2). The liquid-handling arm (LiHa) with 4 fixed steel needles and 4 needles with single-use tips allows liquid level detection through conductivity (Fig. 2b). The robotic manipulator arm (RoMa) with gripper enables labware transfer between the different devices (Fig. 2c). A refrigerator (Revco, Waltham, MA, USA) is used for the storage of highly purified water as system liquid (Water For Injection WFI, Lonza, Basel, Switzerland). Additionally, the platform contains a worktable of 2 m length, pumps for precise liquid transfer, a dark chamber (TIG, Tecan, Crailsheim, Germany) for sample incubation and a sterile laminar flow cabinet over the platform. The cell culture incubator (37 °C and 5% CO_2_, StoreX500, Liconic, Mauren, Liechtenstein) and a Carousel (CarouselNT, Liconic, Mauren, Liechtenstein) for plate supply are directly coupled with the worktable by a transfer station.

The devices follow the guidelines of the American National Standards Institute (ANSI) and the Society for Laboratory Automation and Screening (SLAS) which recommend automation standards (Meets the Standards ANSI/SLAS 1-2004 through ANSI/SLAS 4-2004 2004). The pipetting volume per needle was limited by 1 ml syringe capacity and could be dispensed with three different possible modes. They were called *Free dispense*, *Wet Contact* and *Dry Contact*. The first one, mostly relevant, occurred in the air while the two other modes took place in contact with liquid and labware, respectively.

The system was controlled by the Tecan software *EVOware Standard*. The configuration of the worktable, the carriers and labwares were integrated in the software interface to program processes. Each device was configured in accordance with the characteristics given by the manufacturers. For example, the pre-coated 96 well plate (Mabtech, Nacka Strand, Sweden) has the following specifications: a membrane area of 0.26 cm^2^ and plate dimensions of 127.8 mm × 85.5 mm × 14.4 mm (L × W × D). The program also requires information for both arms, such as the dispense height (1 cm above the membrane), the lowest aspirate position (z-max) without touching the membrane (≤1 mm gap) or the transfer vector between two devices as references. The system was configured to reach 1/10 mm motion and 1–1/10 μl volume accuracy, depending on the tip capacity. The aspirate and dispense commands were defined in “liquid classes”, where flow speed (in μl/s) and air gap (in μl) parameters influence the correct and accurate treatment of the liquid, based on its conductivity and viscosity.

## Results

### Initial preprocessing configuration and validation of the automated procedure

#### Gravimetrical analysis

An important requirement of an automated, reproducible ELISpot-system is the accuracy and precision guaranteed by the tips used for liquid distribution. To determine the appropriate pipette form, we compared in a gravimetrical analysis two different pipetting systems. The results showed that fixed tips enable in general more accurate and reproducible liquid handling with for example −1.029% accuracy against −1.598% for the disposable tips in a 100 μl volume range (Table 1). Due to this results and the plate design, fixed tips were used for cell handling with the *Free dispense* mode and the multichannel and multipipette options resulting in fast, reproducible and contactless suspension transfer. Despite the higher dispersion, the disposable tips were suitable with accuracy under 10% in a 10 μl range. Additionally, they showed a better precision of the liquid distribution in this volume range due to smaller tip capacity (200 μl) instead of 1000 μl for the fixed tips. According to this and especially in order to avoid peptide carryover and time-consuming washing steps in between, the disposable tips were used for the distribution of the three peptide mixes and the control reagent as T cell stimuli for cytokine secretion. It was most important to load the plate with minimal delay to limit the intra-assay variability. With this combination, the automated procedure for plate loading was faster (3 min processing time) compared to the manual procedure (10 min), in which wide bore tips are used for the cells only once per well.

**Table 1:** Gravimetrical analysis of the Tecan Freedom EVO 200

Tips	Pipetted volume (μl)	Mean (μl)	Δ_pipetting_ (%)	CV_pipetting_ (%)
1000 μl Fixed tips (4×) (n = 48)	100	98.971	−1.029	0.320
	10	10.367	3.671	2.307
200 μl Disposable tips (2×) (n = 24)	100	98.402	−1.598	0.585
	10	9.152	−8.483	1.819

Twelve dispensing actions of four fixed tips and two disposable tips are measured with 10 and 100 μl volume on scales. Shown are the pipetting accuracy (Δ_pipetting_) and coefficient of variation (CV_pipetting_) of the system for different pipetted volumes

In addition, selection and adaptation of specific liquid classes were also relevant for the pipetting accuracy. These liquid classes are liquid-dependent, based on conductivity and viscosity characteristics. They consist of a set of parameters such as flow speed and air gap conditions, influencing accurate and precise pipetting liquids. In previous tests (unpublished work) liquid handling parameters were optimized with respect to homogenous microcarrier distribution over a 96 well plate (75% of the wells had an aberration lower than 20%). Based on these experiments, 600 μl cell suspension were mixed 3 times with a moderate dispense flow rate (400 μl/s) at the bottom of the cell container using 1000 μl fixed needles. The inner diameter of the fixed needles (0.5 mm) was smaller than the inner diameter of the 1000 μl disposable tips (0.8 mm). To avoid nonspecific immunological activity induced by shear forces, the donor cells had in effect to be treated carefully and were aspirated with a low flow rate (100 μl/s) for the plate loading. In comparison to the cells, reagents were aspirated with a standard flow rate of 150 μl/s and dispensed with 600 μl/s in plate. A high breakoff speed (400 μl/s) guaranteed blow out of the complete liquid volume, without residues on the tip end.

#### Determination of the tips accuracy

**Fig. 3:**
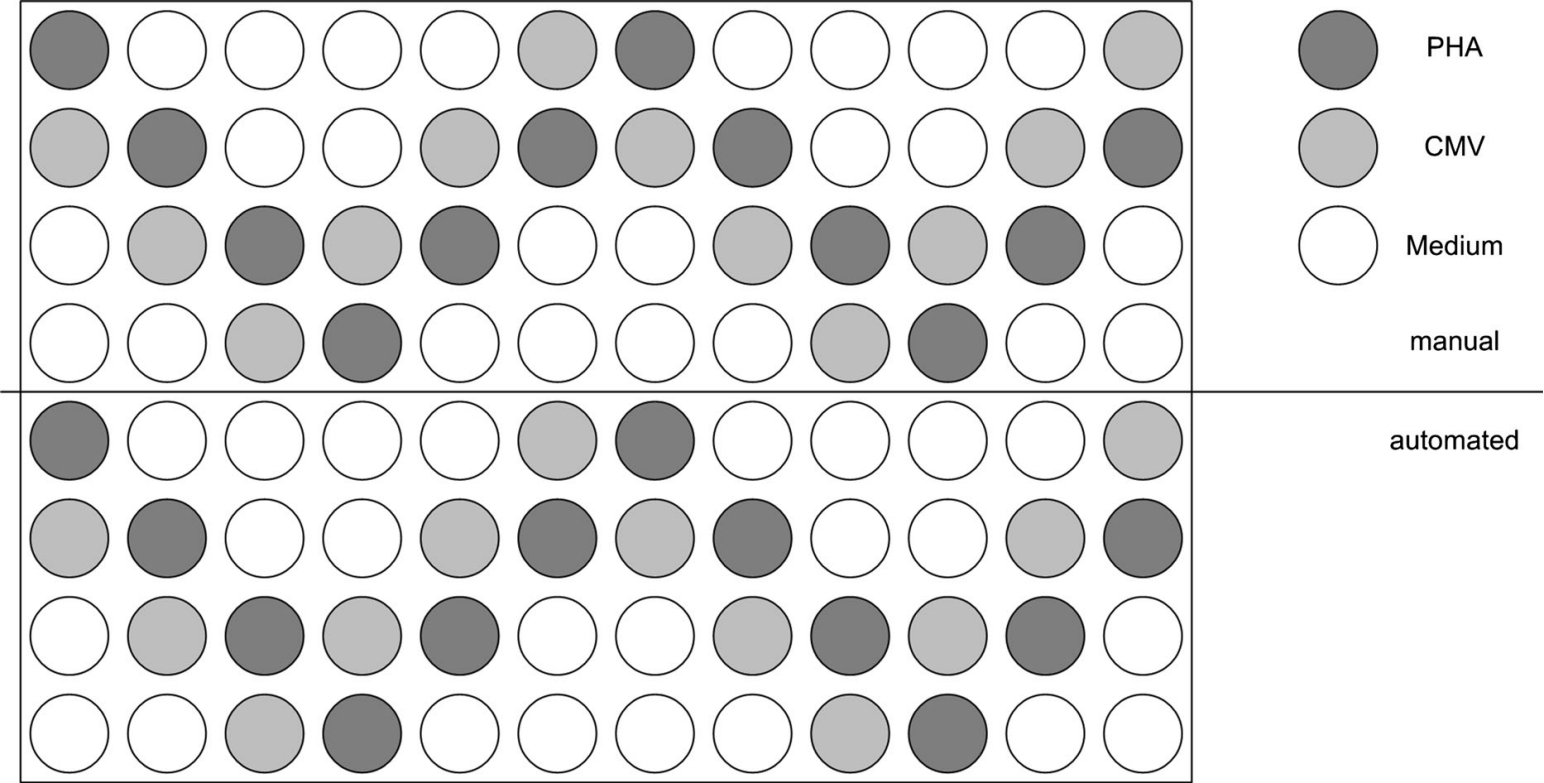
Pipetting scheme of the inter-plate reproducibility test for manual and automated cell adding. The test is performed with manual (*upper part*) and automated (*under part*) cell adding over one half plate and investigates the relation between wells and locations on plate. The wells on the scheme represent the repartition of the manual pipetted PHA (*dark gray*), CMV (*bright gray*) and Medium (*white*)

To confirm the accuracy of the automated pipetting, three donors were tested with each on one half of a plate with the CMV peptide pool and PHA pipetted in W-form over 4 rows (Fig. 3). This test adapted from Almeida et al. (2009) demonstrated the reproducibility of the automated cell adding pipetting step within a plate, medium was used as background negative control. Within general <20% variation, no relation between wells and locations on plate were detected. Values, which were lower than 25% of the mean, were not considered in the calculation. Only one (Second donor with PHA combination in the automated assay) of seventy-two measurements was excluded based on this criteria. The mean CV_spot_ was 11.7% for the manual and 15.9% for the automated assays. The mean Δ_spot_ over the three plates between both pipetting modes indicated a difference of −5.9% for the automated results, which is acceptable.

#### First comparison of the manual and automated procedures (test of the plate loading)

After gravimetrical analysis and pipetting accuracy test (W-Form), we started the comparison of the manual and automated processes. In the first step we focused on the homogenous and reproducible plate loading with reagents and cells referring to *Test 1*. We compared this manually or automatically performed step on four donors in a separate analysis, the rest of the assay was done manually. In the medium-containing part of the automated plate (negative control) no carryover was observed with maximum 1 spot per well. This result confirmed the correct utilization of the *Free dispense* mode (no liquid in well touched during distribution). To be able to compare both assays, Δ and Dis were observed, respectively, as parameter for the relative difference to the manual value and as parameter for range/spreading between both assays. Each sample was measured in triplicates (n = 3). The smaller the Δ and Dis values were, the more precise and comparable were the manual and automated assays. The results of this initial experiment (Table 2, *Test 1*) show a high spread between the manual and the automated plates with a Δ range between 3.7 and 54.5%. For the donor with the highest value (54.5%, donor 1), Dis of the manual assay reached 0.6 and, respectively, 0.4 for the automated assay. In comparison, the donor with the lowest Δ value (3.7%, donor 4) reached a Dis value of 11.5 (manual) and 23.7 (automated). Even in the first step with discrepancy in manual and automated values, a well to well analysis (n = 12) with the Wilcoxon signed-rank test resulted in a *p* value >0.05 and showed no significant difference between both assays.

**Table 2:** Statistics of the manual and automated ELISpot assays for *Test 1* and donor 1 to donor 4

Assay	Donor	Peptide	Δ (%)	Dis_Manual_	Dis_Automated_
Test 1 (Cells and reagents loading)	1	CEF	54.5	0.6	0.4
	1	CMV	10.0	9.2	8.9
	2	CEF	14.3	3.8	9.0
	2	CMV	37.5	0.2	7.5
	3	CEF	11.8	5.1	25.6
	3	CMV	CMV(–)	–	–
	4	CEF	16.4	10.9	0.7
	4	CMV	3.7	11.5	23.7

For each donors (1–4) and the peptide CEF and CMV, Δ and Dis (manual and automated) were evaluated for *Test 1*. The Δ value is representative as accuracy measurement for the relative difference between the manual and automated spot count mean. Dis is referring as a spot distribution statement

### Optimization and enhancement of the automated procedure

#### Technical implementation of the automated process

In order to improve process automation, different parameters were primarily adapted from *Test 1*: cells were mixed only two times and the delay after aspirate action was raised to 300 ms in order to balance the under-pressure in the tips and thereby rest the cells.

**Fig. 4:**
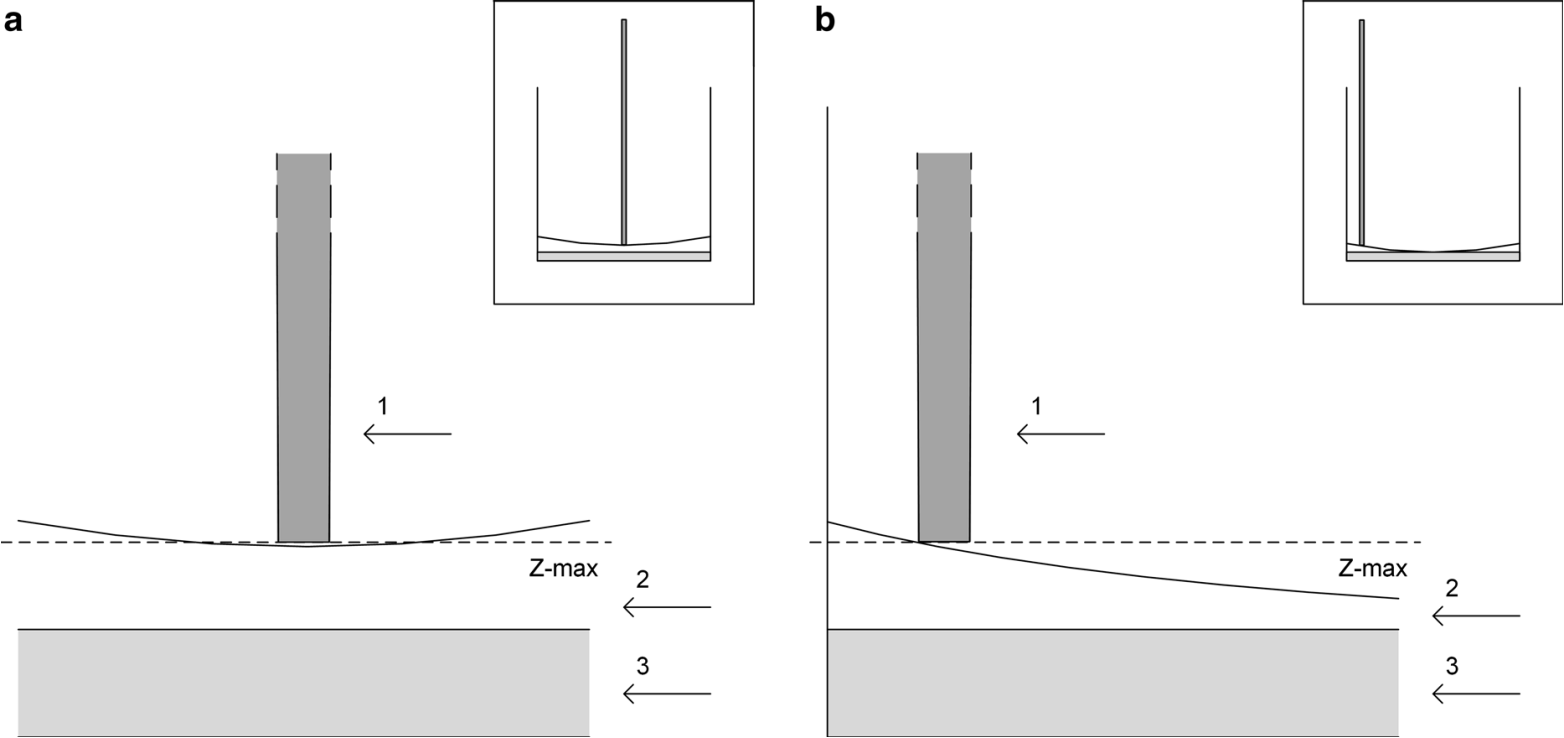
Volume removal from a well with two different approaches. In both cases, the liquid forms a concave level due to membrane properties. **a** fixed tip (1) is positioned in the middle of the well and aspiration is limited by z-max over the whole well length. **b** the tip removes the maximum volume (2) on the edge of the well without touching the membrane (3)

In the following step (*Test 2*) the automated washing steps and antibody dispense were added to the previous reagent and cell loading. Additionally, different parameters were adapted and improved for *Test 2* based on the experience in *Test 1*. PBS aspirate and dispense speeds were, respectively, set to 100 and 200 μl/s instead of 150 and 600 μl/s to avoid long-term damage of the plate membrane. A random liquid increase in the testing plate was observed after repeated washing steps. To counteract this effect, two remedial actions were established. First, the aspirated volume during washing steps was increased from 200 to 230 μl in order to avoid surplus volume. Second, the lowest aspiration position, determined by the constant z-max, guaranteed comparable start volumes in the well for the antibody dispensing step. In accordance with the manual operating procedure, the plate was washed five times with PBS after overnight incubation as well as after antibody incubation. PBS also had to be removed as thoroughly as possible without touching the membrane to avoid dilution of the antibody solution. Normally, any remaining solution is flicked manually onto a paper towel. In the automated process, the way to aspirate liquid during washing steps was changed. We decided not to aspirate the solution from the middle of the well as usual but 2 mm away from the edge. Since liquids build a concave surface due to the protein-binding properties of the plate membrane, more liquid can be removed from the same aspirate height (as shown in Fig. 4). This minimizes the residual volume first estimated as not significant for the following test.

In experiment “*Test 2”*, the results of three combined automated workflow steps (reagent and cell loading, washing steps as well as antibody loading and incubation) were compared to the manual assay. Four donors were tested, one CEF-negative and CMV low-responder (donor 5), one CMV-negative and CEF low-responder (donor 6) and two donors (donor 7 and 8) with an immunological reactivity ranging between 2000 and 7000 SFCs/10^6^ PBMCs. Samples were still run and evaluated as triplicates except donor 8 for CEF reagent, that was evaluated as duplicate due to an outliner. Samples of both negative donors (donors 5 and 6) together with the medium-containing wells showed as expected a low background signal with maximum 3 spots per well. To enable a representative analysis of the further tests, we evaluated only the results of donors 7 and 8 as high and middle responders (Table 3, *Test 2*). Samples from these remaining donors showed a Δ range of 4.2 to 12.1% between the automated ELISpot assay and the manually-performed assay. The changes between *Test 1* and *Test 2* enhanced a decrease of the maximal Δ value of over 75% (54.5–12.1%). The results showed also an amelioration of the Dis_automated_ with a maximal value of 7.5 compared to the manual value of 15.2 and the maximal automated value of *Test 1* with 25.6. The Wilcoxon signed-rank test (n = 12) revealed with a *p* value >0.05 that the automated assay was not significantly different than the manual assay.

**Table 3:** Statistics of the manual and automated ELISpot assays for *Test 2* to *Test 4* and donor 7 to donor 8

Assay	Donor	Peptide	Δ (%)	Dis_Manual_	Dis_Automated_
Test 2 (Addition of washing steps and antibody loading)	7	CEF	6.6	6.0	7.5
	7	CMV	12.1	1.2	1.7
	8	CEF	7.5^a^	5.6	0.1^a^
	8	CMV	4.2	15.2	3.2
Test 3 (Compensation of the antibody concentration)	7	CEF	4.5	0.9	8.7
	7	CMV	4.5	5.8	0.7
	8	CEF	13.9	0.4	18.7
	8	CMV	6.3	1.4	0.4
Test 4 (Addition of the blocking and substrat development steps)	7	CEF	5.8	11.5	1.4
	7	CMV	3.7	12.2	0.7
	8	CEF	6.1	3.3	11.1
	8	CMV	0.7	1.3	4.2

For each donor (5–8) and the peptides CEF and CMV, Δ and Dis (manual and automated) were evaluated for *Test 2*, *3* and *4*. The Δ value is representative as accuracy measurement for the relative difference between the manual and automated spot count mean. Dis is referring as a spot distribution statement. Donors 5 and 6 were tested as controls for *Tests 2*–*4* but are not shown in Table 3 due to their properties as negative CEF and CMV donors, respectively

^a^Values obtained from duplicate instead of triplicate due to one outlier in the automated assay

#### Reagent adaptation based on automation requirements

To further improve the automated process, we introduced for *Test 3* inner and outer ethanol incubations of the fixed tips before and after experiments improving the precision of the volume dispensing and minimizing the adhesion of residual liquid at the tips. Furthermore, owing to the displaced aspirate position during the PBS washing steps, a reduction of residual liquid volume by almost 50% with 14.4 ± 3 μl instead of 28.4 ± 3 μl per well was achieved. Calculated from a safe gap of minimum 0.45 mm between the membrane surface and z-max height of the tip, a residual volume of 13 μl is necessarily remaining in the well. However, this undesired liquid can dilute the antibody solution. To compensate this, the antibody concentration of the automated assays was increased (around 1:155 instead of 1:200 stock dilution) with the final volume remaining at 50 μl per well. The experiment for validating the antibody compensation (*Test 3*) did not show a significant change of the Δ and Dis values, the maximal Δ value being 13.9% compared to 12.1% for *Test 2* (Table 3). The mean Δ decreased slightly between *Test 2* (7.6%) and *Test 3* (7,3%). The Wilcoxon signed-rank test shows that the manual and automated assays were not being significantly different (*p* > 0.05). *Test 3* did not afford a high improvement of the automated process considering the Δ and Dis values. Nevertheless the compensation of the antibody concentration remained in the further step to be able to compare the following results and to ensure an equal antibody end-concentration in the wells between the manual and automated assays.

### Final proof of concept

Based on the experiences gained in *Tests 1, 2* and *3* many factors have been improved altogether for the automated process. Complementary optimizations of the process have also been introduced for *Test 4* with the reduction of dead and cache volume. Due to liquid handling parameters such as air gap, surplus of reagent and cell solutions were prepared. To avoid wastage, the minimum required volume in the reservoir for cells or reagents before aspiration has been characterized. The supply vessel used for these experiments has an inner diameter of about 6 mm and a safety gap (between vessel base and lowest aspiration position) was set to 1 mm. The dead volume of the container was about 30 μl (π × R^2^ × H = π × 3^2^ × 1 = 28.3 μl + minimal volume due to conical form of the vessel ≈ 30 μl). During the process 20 μl were lost through multiple pipetting actions. Hence, the cache volume must be around 10% of the needed volume or at least more than 50 μl (=minimum volume in cryovial + process loss volume). This value is equivalent to the conditioning volume used in standard multi-pipetting devices. The conditioning volume corresponds to the volume dropped back to the reservoir vessel before pipetting and guarantees an accurate volume dispensing.

**Fig. 5:**
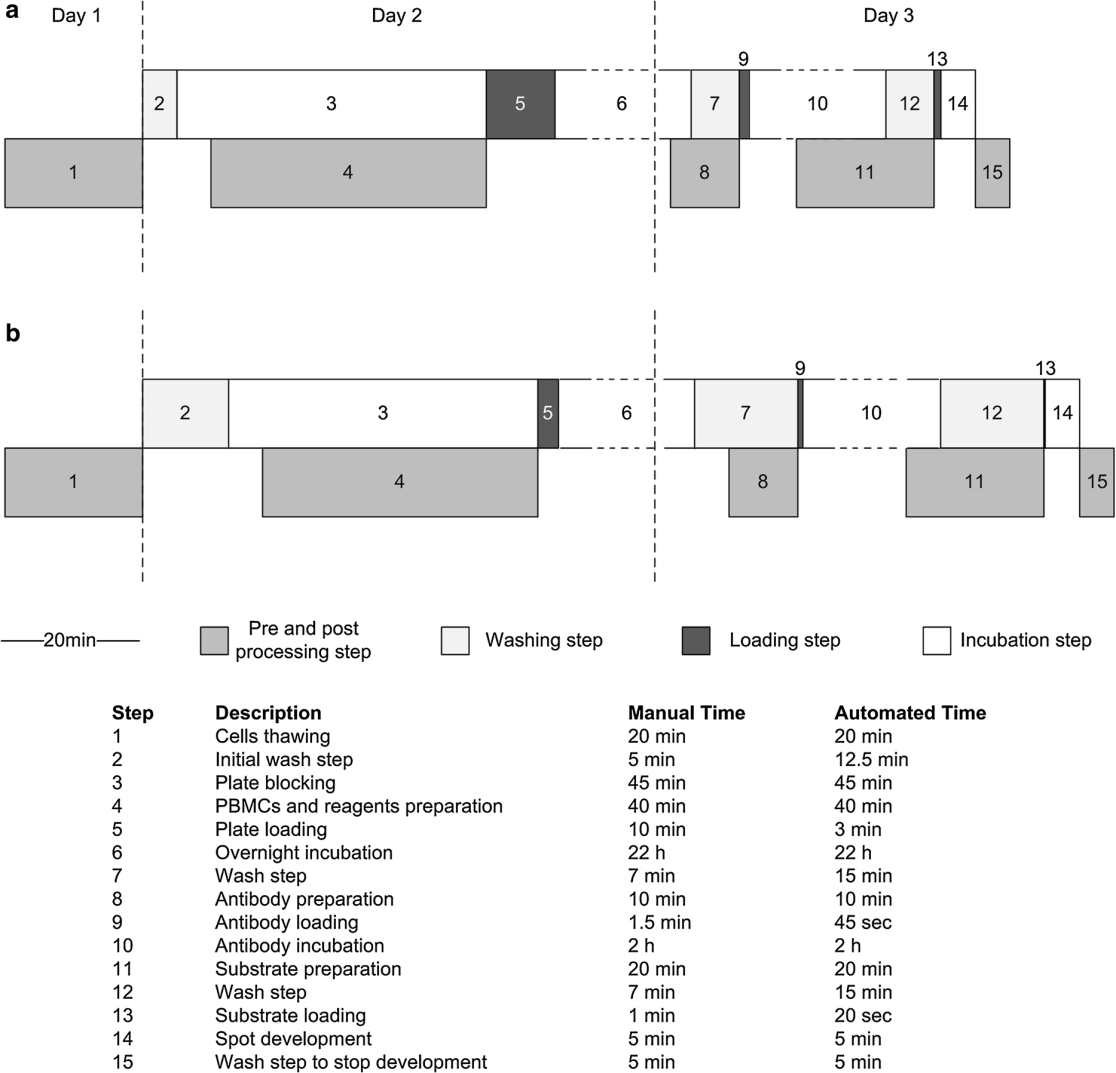
Timeline of the manual (**a**) and the new established fully automated (**b**) ELISpot assay. The preparation of cells, reagents, antibodies and substrate as well as the plate post processing (*dark gray*) still occurs manually and parallelized to the automated protocol. The *bright gray* parts show the washing steps that take longer for the automated assay compared to the black parts illustrating the loading actions, which are faster. The *white boxes* indicate the incubations and the *scale* represents 20 min process time. In the joined table, real times of each assay operation (steps 1–15) are enumerated for both modes

Many parameters had to be considered to automate the manual ELISpot assay. As resumed and shown in Table 4 the accumulated optimization steps were focused on pipetting accuracy, washing step conditions and avoidance of carryover. Considering all these factors, the last two steps of the assay, plate blocking and substrate development, were automated (Test 4) and added to the process as illustrated in Fig. 5. The workflow represents and enumerates all steps of the manual versus automated assay with the corresponding processing duration, some preparation steps still having to be done manually. As revealed in Fig. 5, the automated assay was still about 15 min longer than the manual assay due to the exhausting washing steps. Nevertheless, the critical loading steps were 2–3 times shorter.

**Table 4:** List of encountered problems during the establishment of the ELISpot assay on our automated system and the measures taken to improve the process to the level of the manual assay or beyond

Occurred problems	New parameters	Optimization
Dripping tips	High blow out speed (400 μl/s)	✓
Carrying over due to air bubbles at the wells’ top	Z-dispense set to about 5 mm above well	✓
Volume elevation after washing steps	Increased removed volume (230 μl instead of 200 μl)	✓
Remaining volume in wells	Aspiration of liquid on edge of well	✓
Long-term damage of cytokine-binding membrane during washing steps	PBS aspirate and dispense speeds reduced to 100 μl/s and 200 μl/s, respectively	✓
Residual liquid at tip end	EtOH wash before and after use	✓
Dead volume in 96 well plate	Increased reagents and antibody concentration for the equal pipetted volume (50 μl)	✓
High reagent consumption	Cache reduced to 10% (at least 50 μl)	✓

**Fig. 6:**
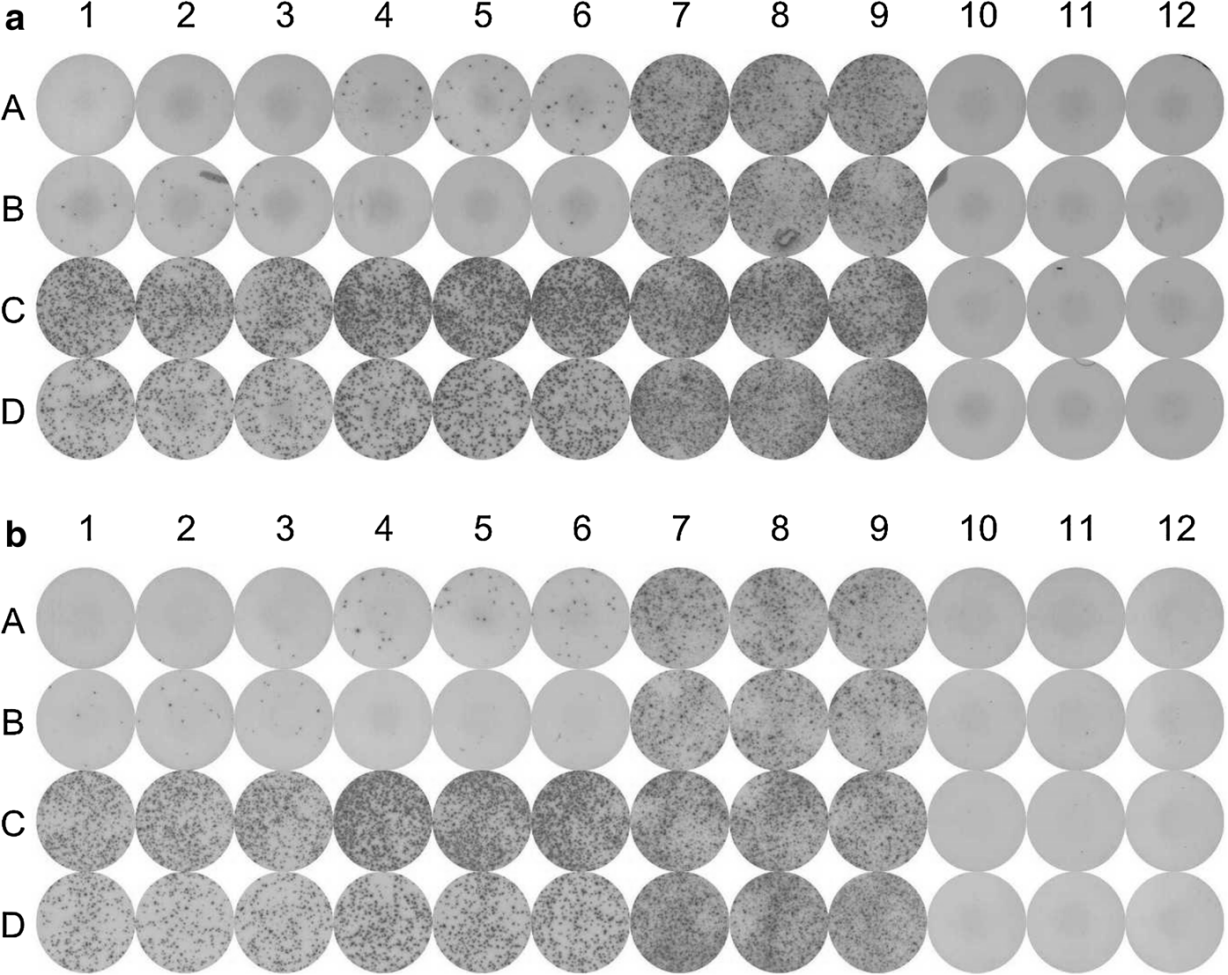
Comparison of manual (**a**) and automated (**b**) ELISpot plates tested with four donors (Lines A–D, donors 5–8). Columns 1–3 show the secreting cells stimulated by CEF, columns 4–6 the ones stimulated by CMV, columns 7–9 by PHA and columns 10–12 by medium. The amount of counted spot forming cells (SFC) represents the immunoreaction of each donor. The scans were operated and evaluated on an automated CTL ImmunoSpot plate reader

**Fig. 7:**
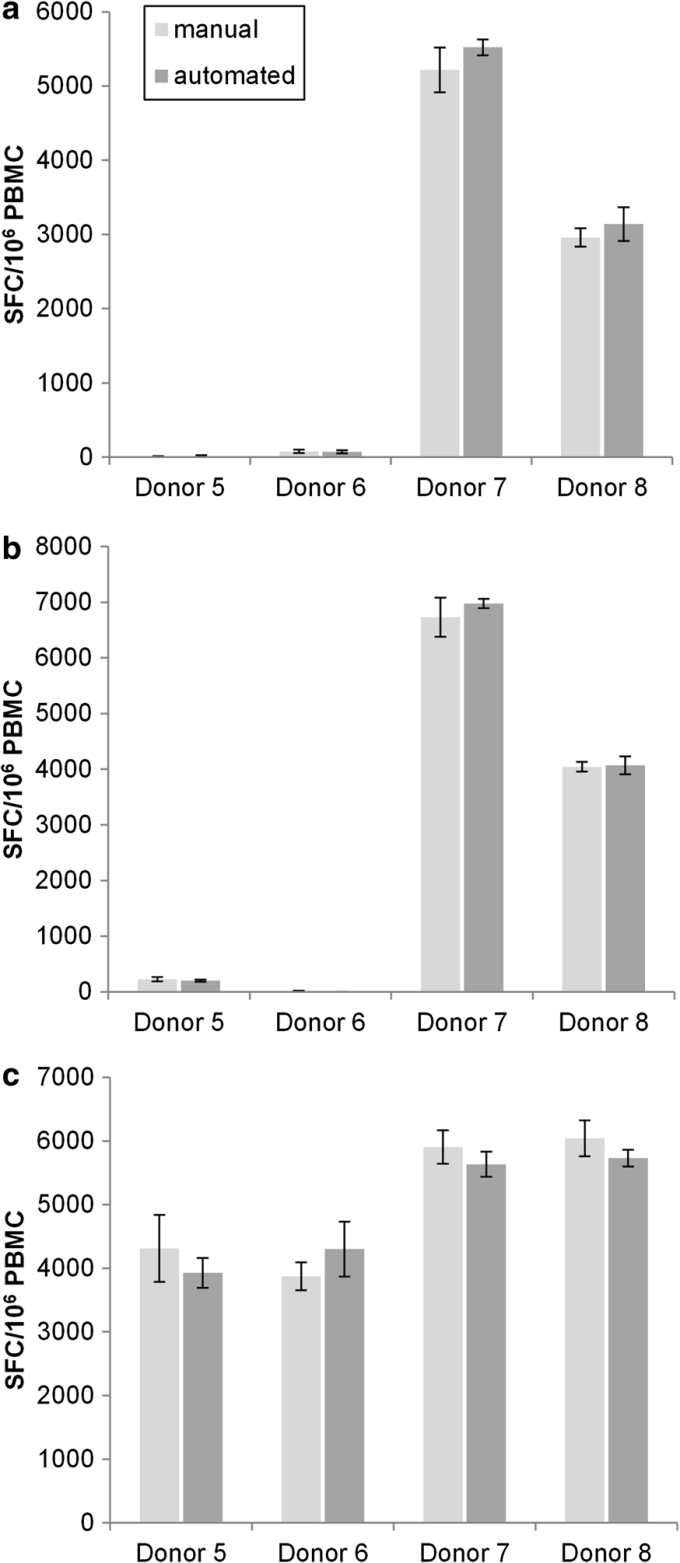
Comparison of the spot forming cells (SFC) amount per 10^6^ added PBMCs for four donors in manual (*bright gray*) and automated (*dark gray*) ELISpot assay. The histogram illustrates the results of Test 4, comparing the manual and the automated spot counts. **a** represents the cells reaction with CEF reagent, **b** the reaction in presence of CMV and **c** shows the positive control with PHA. Donor 5 is CEF negative and CMV low-responder and Donor 6 CMV negative and CEF low-responder. Both donors are used as carryover and selectivity controls. Donor 7 and 8 are considered as high and middle responders with reactivity between 2000 and 7000 SFCs/10^6^ PBMCs

For the final proof-of-concept, we compared the automated ELISpot assay including following steps: blocking, cell and reagent loading (compensated) antibody loading, washing steps and substrate development. The optimized automated ELISpot assay was evaluated using the same donors (5–8) than in *Test 2* and *3* (Fig. 6). Two plates are exemplarily shown after spot development: one was prepared completely manually (Fig. 6a), the other one with the automated ELISpot assay in parallel (Fig. 6b) with the plate remaining in the system during the complete process. For a more representative view of the comparison, a histogram has been produced to illustrate the results. Figure 7 shows the amounts of spot forming cells (SFC) per 10^6^ PBMCs for the reagents CEF, CMV and PHA of the four donors (5–8) in the manual and in the automated assay. The results of Test 4 show a Δ range of 0.7–6.1% (Table 3, *Test 4*). This is a decrease of the maximal Δ value of over 50% compared to Test 3 (13.9%) and of over 88% compared to Test 1 (54.5%). Dis_manual_ has a higher maximal value with 12.2 than Dis_automated_ with 11.1. Dis_automated_ mean is also smaller than the manual one. The well to well analysis with the Wilcoxon signed-rank test results in a non-significant difference between the manual and the automated assays (*p* > 0.05).

Finally, the automated ELISpot assay reached, through process improvements described here, an acceptable Δ mean of maximum 6% with a Dis mean smaller than in the manual assay. In contrast to CEF and CMV (with negative donors), PHA values were analyzed for all four donors (5–8). During the implementation phase, same donors were used for *Test 2, 3* and *4* allowing an intra-assay comparison of the manual plates. Manual delta difference of *Test 2* against *Test 3*, *Test 2* against *Test 4* and *Test 3* against *Test 4* were calculated per donor. The mean of the three correlations or comparative values was taken resulting in an average of 26.2% showing the high intra-assay variability of the manual procedure. This value was important and has been used as reference value in order to rank the inter-assay values of the comparison between the manual and the automated process. An intra-assay comparison of the automated plates was not possible due to step by step improvement of the automated assay conditions between Test 2, 3 and 4.

In the case of the inter-assay consistency, a big enhancement was observed in the comparison of the Δ between manual and automated plates over the implementation process for the PHA values. For *Test 1*, the mean of the Δ value was 25.3%, for *Test 2* 22.0%, for *Test 3* 15.8% and for *Test 4* 7.4%. The results are listed in Table 5 and show a three times smaller inter-assay inconsistency between the manual and the automated processes of the improved test (*Test 4,* 7.4%) than the manual mean intra-assay inconsistency (26.2%).

**Table 5:** Statistics of the inter- and intra-assay inconsistency for *Test 1* to *Test 4* and donor 1 to donor 8 for the PHA reagent

	Comparison	Δ Mean (%)	Mean
Intra-assay consistency (manual)	Test 2 against Test 3	31.9	26.2
	Test 2 against Test 4	38.1	
	Test 3 against Test 4	8.7	
Inter-assay consistency (manual against automated)	Test 1	25.3	–
	Test 2	22.0	–
	Test 3	15.8	–
	Test 4	7.4	–

The mean delta value represents the mean of the Δ between the manual and automated spot count mean for the four donors

## Discussion

The ELISpot assay is one of the most important techniques for immunomonitoring purposes and vaccine development. As a central method involved in international HIV research projects and clinical trials, the assay has to be conducted at a high level of standardization and reproducibility worldwide to guarantee comparability of results (Janetzki et al. 2005). The most significant challenges are its inter-operator as well as inter- and intra-assay inconsistency. To address this problem, we developed an automated ELISpot assay based on a liquid handling pipetting platform, minimizing variability errors caused by human and unstandardized factors. We focused our investigation on (1) adapting the manual procedure, (2) optimizing liquid classes for plate loading, (3) minimizing dead volumes and (4) adapting reagent consumption and concentrations.

We were able demonstrate an implementation of the manual ELISpot process on the automated platform with comparable ELISpot counts and more homogenous results than in the manual assay.

The results presented here are a first proof of principle of an automated ELISpot procedure. Aside of the technical issues of the process automation, different challenges have been solved to enable the transfer of the ELISpot assay on the platform. One issue was the reduction of the dead volume cumulated during washing steps resulting in assay variability. Due to the solution of the adjusted peripheral aspiration even up to 14.4 μl are still left, requiring to concentrate the antibody. A complementary limitation was the significantly increased time needed for the automated washing steps in comparison to the manual procedure. Indeed, the manual process includes plate flicking that could not be transferred completely to the automated system described here. However, in all highly critical actions, the automated process was at least two to three times faster than the manual process (e.g. automated plate loading could be reduced to 3 min in contrast to 10 min for the manual loading). For future improvement of the process, an automated washer or a 96 liquid handling head instead of the LiHa could be implemented in the routine to accelerate the washing step. This head is based on a complex multichannel system able to operate a plate in total so that multiple pipetting steps become redundant. Additionally, the velocity of plate loading and washing will increase significantly as the process of aspirating and dispensing a 10 μl volume in each well of a 96-well plate will only last 25 s (as specified by the manufacturer), resulting in more homogeneously and continuously processed plates per 24 h.

Technical limitations aside, biological effects have also been controlled. To check the automated process on cross-contamination and selectivity, negative CEF/low-responder CMV and negative CMV/low-responder CEF donors (donors 5 and 6) were used in *Test 2*. The same donors were implemented consistently in *Test 3* and *Test 4* due to comparability. No carryover was observed throughout all experiments.

Here we prove for the first time that the automation of main ELISpot assay steps in a robotic platform is possible and comparable to the manual standard protocol. Advantages of this automated assay are standardization, improved safety, reproducibility, quality control, and scalability as has previously been suggested by others (Franscini et al. 2011). In automated routines plates can be processed without human assistance. It is possible to schedule plate processing using a nested process structure resulting in an optimal workflow timing, working day and night. An automated system, being more accurate and reproducible than an operator, will reduce the intra- and inter-assay variability to its minimum, for example regarding the continuously control of incubation time (Allinson 2011). Compared to a technician processing four to six plates within one working day, the automated device can handle up to twelve plates in the same time with the technician focusing on other work in parallel. Altogether the turnaround time with an automated ELISpot assay is about four to six times higher than manual performed ELISpots. Our results directly address the growing need for (integrated) automation in cell processing and storage as robotic systems can improve accuracy and precision as well as time- and cost-efficiency (Bodin 1995; Cox et al. 2004; Dimech 2000).

The robot platform is highly flexible and programmable for different purposes. It has already been configured and applied for e.g. stem cell differentiation (Meiser et al. 2013) and for the cultivation and maintenance of different cell lines, like pluripotent stem cells. To extend the platform, a centrifuge, a reagent cooling system as well as an optional coupled cryopreservation station could be added in the future, enabling the complete assay to be run automatically from PBMC isolation over cryopreservation, cell and reagent preparation to plate development. This complex scenario matches the vision of Janetzki et al. (2004) a decade ago and since required hardware is now available it could be developed in the near future. Devices for automated blood isolation (PBM200, AM Robotic Systems, Warrington, United Kingdom) and semi- and fully automatic freezing and thawing supplies are now commercially available (Immunocite Technologies, Miramar, FL, USA, and MéCour, Groveland, MA, USA) according to the standards of the Society for Laboratory Automation and Screening (SLAS). Besides, automatable cell preparation tubes (Vacutainer CPT, BD, Heidelberg, Germany) are used as improved alternative to Ficoll gradient (Ruitenberg et al. 2006) for PBMC separation. The platform could be used after validation as a ready-to-use and fully automated cell and liquid handling system with the integration of a 96 multichannel head, the cell separation system including a centrifuge, and the adaptation of a cryo storage supply. This system would have the ability to produce high-throughput, safe and GLP-conform ELISpot assays that are required in clinical trials (Slota et al. 2011) as well as in laboratories with high biosafety levels in which research for instance on tuberculosis (Kobashi et al. 2010), malaria (Gonzalez et al. 2000) or Bulgarian Crimean–Congo hemorrhagic fever (Mousavi-Jazi et al. 2012) demand secured tests. With the technical potential and the capacity for adaptations, the robot platform could not only be used for stem cell research and immunological assays such as neutralization tests for HIV vaccine search (Schultz et al. 2012) but also for multiple further applications where accuracy, precision, reproducibility, scalability and operator safety are crucial.
